# Digital Phenotyping and Noninvasive Quality-of-Life Monitoring in Glioblastoma: Feasibility and Usability Study of the Lalaby-Glio App

**DOI:** 10.2196/89765

**Published:** 2026-10-01

**Authors:** Sabina Asensio-Cuesta, Elies Fuster-García, Juan M García–Gómez, Carlos Sáez, Daniel Sánchez–García, Ángel Sánchez–García, Jorge Soler, Inmaculada Maestu, Teresa Soria-Comes, Maria De Julian Campayo

**Affiliations:** 1 Universitat Politècnica de València Instituto de Tecnologías de la Información y Comunicaciones Valencia Spain; 2 Consorcio Hospitalario Provincial de Castellón Castellón Spain; 3 Hospital Universitario Doctor Peset Valencia Spain

**Keywords:** digital phenotyping, feasibility, glioblastoma, mobile health, passive sensing, patient-reported outcome measures, PROMs, QoL, quality of life, usability

## Abstract

**Background:**

Glioblastoma multiforme (GBM) is the most common and aggressive primary brain tumor in adults, with a poor prognosis despite Stupp protocol treatment. Given the substantial burden of the disease and its therapies, monitoring health-related quality of life (HRQoL) is essential. Smartphone-based approaches may enable continuous, low-burden monitoring.

**Objective:**

This study aims to evaluate the feasibility and usability of the Lalaby-Glio app for monitoring HRQoL in patients with GBM and to explore preliminary hypothesis-generating associations between subjective global quality of life (QoL) and passive smartphone sensor data.

**Methods:**

The study comprised four phases: (1) adaptation of Lalaby for GBM-specific monitoring by combining passive smartphone sensors (movement, step count, location, sound, light exposure, internet use, and call activity) with a clinician-designed daily 5-item questionnaire and weekly European Organization for Research and Treatment of Cancer Quality of Life Questionnaire-Core 30 (QLQ-C30) and Quality of Life Questionnaire-Brain Neoplasm 20 (QLQ-BN20); (2) a 6-week feasibility study in 4 adults (aged ≥18 years) initiating Stupp treatment; (3) usability testing in patients with GBM and a complementary nonpatient sample of 103 healthy adults using either screenshot mock-ups or the installed app; and (4) exploratory Spearman correlation analyses of associations between passive sensor data and subjective global QoL using patient-level bootstrap, with no causal or predictive claims inferred due to the small sample size.

**Results:**

We enrolled 4 patients (3 men, 1 woman; mean age 54.3, SD 8 years), of whom 3 completed all 6 weeks, and 1 died after 2 weeks. Engagement was high, with 168 questionnaires completed (22 QLQ-C30, 19 QLQ-BN20, and 127 daily status entries). Mean app use was 1.8 (SD 2.7) minutes per day, and mean completion time for the weekly questionnaires was 7.2 (SD 3.3) minutes. Weekly patient-reported outcome measures (PROMs) revealed substantial interindividual and intraindividual variability in functioning, symptoms, and global QoL during treatment. Usability among patients with GBM was favorable (overall mean 3.7/5). Among healthy participants (mean age 20.9, SD 1.4 years; 72/103, 69.9% women), those who installed the app (n=46) achieved a mean System Usability Scale score of 72.9 (SD 10.2), above the standard benchmark, and 91.3% (94/103) judged the nature-inspired design appropriate for oncology. Passive sensing yielded 91.7 MB of data; global QoL correlated negatively with calls (ρ=−0.598; *P*=.01) and movement (ρ=−0.604; *P*<.001), while steps showed a positive but nonsignificant association (ρ=0.392; *P*=.63). These correlations should be interpreted as preliminary signals rather than robust evidence of digital biomarkers.

**Conclusions:**

Lalaby-Glio appears feasible and usable for monitoring HRQoL in patients with GBM in an early proof-of-concept setting and can capture patient-reported fluctuations over time. Passive smartphone sensing shows promise for complementing PROM-based monitoring and generating preliminary hypotheses about objective behavioral correlates of QoL.

## Introduction

Glioblastoma multiforme (GBM) is the most aggressive primary malignant brain tumor in adults, with a median survival of 12 to 15 months [1]. Beyond its limited prognosis, the disease and its intensive standard treatment under the Stupp protocol [2]—maximal surgical resection followed by concomitant radiotherapy and temozolomide and subsequent adjuvant temozolomide—impose a profound functional, cognitive, and psychosocial burden. Consequently, preserving and monitoring health-related quality of life (HRQoL) is a central priority in GBM care, as patients may experience rapid and clinically meaningful changes in daily functioning and well-being throughout the treatment trajectory [3].

Today, cancer treatment aims not only to prolong survival but also to alleviate symptoms and maintain or improve quality of life (QoL). Thus, the benefits of both existing and novel treatments must be weighed against side effects and potential deterioration in patients’ QoL. HRQoL has become an increasingly important end point in cancer studies, alongside outcomes such as overall survival and progression-free survival, particularly in patients with incurable diseases [4].

HRQoL is a multidimensional concept that describes the physical, role-functioning, social, and psychological aspects of well-being and functioning and may incorporate both objective and subjective perspectives within each domain [5]. The primary goal of using and measuring HRQoL is to provide a more comprehensive and valid evaluation of an individual’s or population’s health status and to offer a clearer assessment of the benefits and risks associated with medical care. Standardized HRQoL data are also expected to help improve health care quality [6].

HRQoL is typically included as a secondary end point in brain tumor clinical trials [4,7-12], serving as a key factor in assessing the net clinical benefit of a treatment strategy. This implies weighing potential survival gains against negative treatment effects on functioning and well-being [13]. The most common instruments used in brain tumor trials to assess HRQoL are the European Organization for Research and Treatment of Cancer (EORTC) Quality of Life Questionnaire-Core 30 (EORTC QLQ-C30) [14], the Functional Assessment of Cancer Therapy-General [15], and the MD Anderson Symptom Inventory [16], typically combined with brain-specific modules such as the Quality of Life Questionnaire-Brain Neoplasm 20 (QLQ-BN20) [17-19].

Patient-reported outcome measures (PROMs) and symptom-tracking data have the potential to advance medical understanding and ultimately improve treatment [20]. Mobile apps can offer personalized, real-time monitoring and may address limitations of self-reporting during medical consultations [21], such as recall bias or underreporting of symptoms, which may be exacerbated by cognitive deficits in some patients with GBM [22,23].

In the context of GBM, Ellen et al [20] developed and tested the OurBrainBank app, enabling symptom tracking by patients. In their pilot study with 630 participants (patients with GBM or caregivers), an initial sociodemographic and clinical survey was completed, followed by the EQ-5D-5L HRQoL survey [24]. This tool captures 5 health dimensions—mobility, self-care, usual activities, pain/discomfort, and anxiety/depression—across 5 severity levels and includes a global health rating scale from 0 to 100. QoL was assessed using the EORTC QLQ-C30 and the brain cancer–specific QLQ-BN20. The app supports patients in managing their condition while also enabling anonymized data collection for research. More recently, Nawabi et al [25] introduced the use of digital phenotyping in GBM, focusing on step count, time spent at home, total distance traveled, and number of places visited across the preoperative, immediate postoperative, and late postoperative periods. Using the Beiwe app to passively collect smartphone GPS data, they characterized mobility trends in patients with GBM throughout the course of treatment [26].

However, existing approaches, including Beiwe, have largely concentrated on mobility-related indicators and/or have relied predominantly on self-reported symptoms and standardized HRQoL questionnaires, without fully exploiting the broader range of signals available from smartphone sensors. Lalaby-Glio, an adaptation of the Lalaby app [27], was developed to explore the feasibility of integrating self-reported HRQoL data with a broader set of passive data streams, including movement intensity, ambient light and sound, data use, and screen use. In this study, the term “digital phenotype” refers to a preliminary, patient-level integration of active questionnaire-based data and passive sensor-derived metrics, rather than to a validated predictive model of HRQoL. This integrative approach is intended to provide contextual information on patient status over time and to support hypothesis generation regarding potential relationships between passive behavioral patterns and perceived QoL.

Against this background, we investigated the feasibility and usability of smartphone-based data for assessing HRQoL in patients with GBM. We describe the adaptation of the Lalaby app and dashboard for GBM-specific HRQoL monitoring (Lalaby-Glio), report a 6-week feasibility study in 4 patients with longitudinal HRQoL outcomes, and present complementary usability and user experience (UX) findings from patients with GBM and healthy adults. We also examined associations between passive sensor data and subjective global QoL using Spearman correlations with patient-level bootstrap as an exploratory, hypothesis-generating analysis given the small sample size.

## Methods

### Ethical Considerations

Ethical approval for this study was obtained from the Ethics Committee of the Consorcio Hospital General de Castellón (Act No. 62), the Hospital Universitario Doctor Peset de Valencia (CEIm 08/24), and the Universitat Politècnica de València (approval code P11_26-03-2025; dated March 26, 2025). All participants provided written informed consent prior to enrollment. The study was conducted in accordance with the Declaration of Helsinki and relevant national regulations on clinical research and data protection.

### The Lalaby-Glio App

The Lalaby app was adapted to meet the specific needs of patients with GBM and renamed Lalaby-Glio. This was accomplished by customizing the information used to configure the digital phenotype and modifying the app’s design and visual appearance to enhance the overall UX for this patient group.

To define the information included in the digital phenotype, the existing version of the Lalaby app was used as the basis for passive data collection [27], capturing parameters such as movement, distance traveled, number of calls (incoming and outgoing), sound (frequency and decibel level), and internet usage. Additionally, step count and ambient light (lux) were integrated.

The oncologists participating in the study specified the active data as PROMs used to assess patients’ daily status. These comprised hours of sleep (day and night); frequency of specific symptoms (seizures, nausea, and vomiting), rated on a scale of 1 to 3 or more; headache intensity (“a little,” “quite a bit,” or “a lot”); irritability (“mild,” “moderate,” “intense,” or “very intense”); an option to indicate nonlisted symptoms; and perceived walking ability (“without difficulty,” “alone but with difficulty,” “with help,” or “with a wheelchair”).

For the weekly status monitoring of HRQoL, the standardized EORTC QLQ-C30 and the brain tumor–specific QLQ-BN20 questionnaires were selected. In terms of UX evaluation, based on the clinical team’s recommendations and findings from previous Lalaby pilots, the original feedback mechanisms—free-text and audio input, as well as the User Experience Questionnaire-Short Version [28]—were removed. These were replaced by a single, simplified 1 to 5 rating of the app experience to reduce the cognitive and emotional burden on patients with GBM.

Based on the hypothesis that exposure to nature may provide psychological and physiological benefits for patients with cancer [29-31], this concept was incorporated into the redesign of the Lalaby app for patients with GBM. The visual interface was inspired by the “tree of life” and adapted to reflect elements typical of the Valencia region (Spain), where the study was conducted, including the symbolic presence of orange and lemon trees and color palettes representing the sea, sunlight, and blue skies. These design choices aimed to create a soothing and locally grounded visual environment, potentially enhancing patient comfort and engagement (Figure 1 provides screenshots of the Lalaby-Glio app).

**Figure 1:**
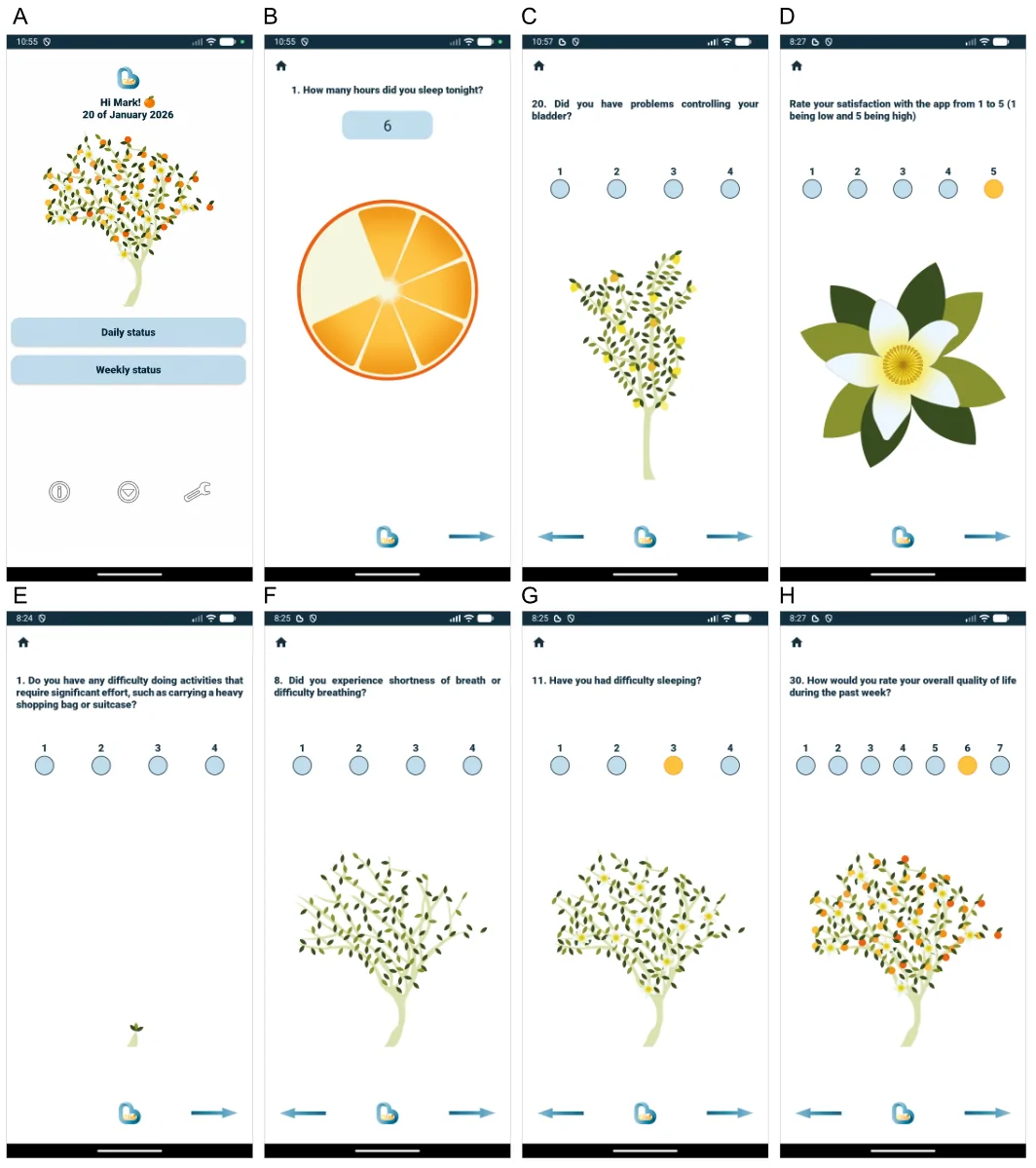
Lalaby-Glio app screenshots illustrating interfaces and questionnaire items. (A) Main menu. (B) Daily status: sleep hours. (C) European Organization for Research and Treatment of Cancer (EORTC) Quality of Life Questionnaire-Brain Neoplasm 20 (QLQ-BN20): item 20. (D) In-app rating (score 1-5). (E) EORTC Quality of Life Questionnaire-Core 30 (QLQ-C30): item 1. (F) EORTC QLQ-C30: item 8. (G) EORTC QLQ-C30: item 11. (H) EORTC QLQ-C30: item 30.

In addition, gamification elements were incorporated into the app to introduce a sense of dynamism and engagement. As patients completed the questionnaires, visual animations displayed the gradual growth of an orange and a lemon tree on the screen, symbolizing their life cycle and reflecting the user’s ongoing participation.

In parallel, the Lalaby dashboard was redesigned to present digital phenotype data in a clinically interpretable format, enabling health care professionals to monitor each patient’s status remotely over time. An integrated alert system was developed with a dual objective. First, from a clinical perspective, it serves to notify oncologists of significant declines in HRQoL, based on predefined threshold values derived from standardized questionnaire scores and reported symptoms within the app. These thresholds were established in collaboration with clinicians to ensure medical relevance. Second, from the perspective of usability and patient experience, the alert system was designed to detect potential digital burdens that may negatively impact the patient’s well-being or engagement. In such cases, and following a joint in-person evaluation between the patient and their oncologist, use of the app may be suspended, reverting to traditional face-to-face monitoring. This approach ensures that the system responds not only to HRQoL indicators established by oncologists for monitoring purposes, but also to the user’s subjective experience when using the app, reinforcing a flexible, patient-centered model of care.

### Recruitment

Participants in the study evaluating the Lalaby-Glio app were adult volunteers who met the following inclusion criteria: aged 18 years or older, diagnosed with GBM, and scheduled to begin standard treatment (Stupp protocol). Participants were required to own a smartphone with the Android operating system (version 4.0.3 or higher, optimally version 7.0) and have access to the internet (Wi-Fi and 3G, 4G, or 5G). All participants had to be informed about the study objectives and provide written informed consent.

Exclusion criteria included individuals who were unable to provide informed consent, as well as patients unfamiliar with the use of mobile apps or those with an incompatible smartphone.

### Study Design and Procedures

The duration of participation for patients with GBM was established at 6 weeks. In the week before the initiation of treatment, oncologists informed eligible patients about the Lalaby-Glio app and the objectives of the study, providing an information sheet and the consent form. Patients who wished to participate were asked to submit the signed voluntary participation form at their next visit.

On the day treatment commenced, the Lalaby-Glio app was installed on the patient’s smartphone during an in-person session. This approach was crucial for building trust in the app [32] and ensuring that patients fully understood the questions and the app’s functionality, thereby minimizing potential burden. This procedure had been previously validated in a prior study involving patients with lung cancer [27,33].

The usability of the app was assessed using the “think-aloud” technique during the face-to-face installation with patients with GBM, as well as throughout a complete usage cycle, which included completing the “daily state,” “weekly state,” and sharing their overall experience with the app. This method allowed for real-time observation of patients’ interactions with the app and provided valuable insights into their thought processes.

Additionally, a questionnaire was prepared to gather feedback on the app’s usability, aesthetics, and overall UX after the first use. The questionnaire included questions about the patient’s mobile device type and Android version, the app’s ease of use, and its aesthetic appeal. Patients were asked to rate the app on a scale of 1 to 5 for its ease of use and aesthetic qualities. They were also asked to share their thoughts on the nature-inspired imagery included in the app, with options for them to indicate whether it had a positive, negative, or neutral impact on their experience.

Valuable usability insights can be obtained even from users outside the app’s primary target group. As Krug [26] highlights, testing with just a few users, even nonspecialists, can reveal most usability issues. In this context, industrial design students offer a valuable perspective: although not oncology patients, their academic training in user-centered design enables them to detect interface issues and suggest improvements. To complement patient-based testing and address the difficulty of recruiting a large sample of patients with GBM, we conducted a supplementary study with this group.

Participants voluntarily interacted with a modified version of the app, specifically adapted for usability testing purposes to ensure that no user data were collected. After using the app, participants completed a questionnaire that included the System Usability Scale (SUS) [34], as well as a question asking them to describe any errors they had encountered and propose solutions. The questionnaire also included additional items assessing the simplicity and visual appeal of the app, and the suitability of nature-inspired imagery for personal use and application in oncology care (Table 1).

**Table 1:** Survey items for evaluating usability and aesthetic perception of the Lalaby-Glio app.

Question	Response scale
Q1: rate the simplicity of the current Lalaby app.	1 (very poor) to 5 (excellent)
Q2: rate the visual aesthetic of the current Lalaby-Glio app.	1 (very poor) to 5 (excellent)
Q3: do you think nature-inspired images influence your user experience?	Positively, negatively, or no influence
Q4: do you think nature-inspired images influence the experience for oncology patients?	Positively, negatively, or no influence
Q5: do you think a nature-inspired design is appropriate for oncology patients?	Yes or no
Q6: why?	Free-text response
Q7: what other themes do you think might be appropriate for the Lalaby app in oncology contexts?	Free-text response

### Statistical Analysis

Exploratory analyses were conducted to examine associations between passive smartphone sensor data and subjective global QoL derived from PROMs. Given the small sample size, nonnormal data distributions, and the presence of repeated measurements within individuals, associations were assessed using Spearman rank correlation coefficients. These analyses were considered exploratory and hypothesis-generating; therefore, no causal, predictive, or definitive clinical conclusions were inferred from the observed correlations.

To account for within-patient clustering of longitudinal observations and to avoid inflation of statistical significance due to the nonindependence of repeated measures, a patient-level bootstrap approach was applied. In this procedure, resampling was performed at the patient level, preserving all repeated observations within each patient in each bootstrap sample. Correlation coefficients, 95% CIs, and *P* values were estimated from the bootstrap distribution.

For ambulatory-related metrics (eg, step count and movement intensity), analyses were repeated excluding the wheelchair user to ensure biomechanical comparability. Accordingly, all correlation results should be interpreted with caution and regarded as preliminary signals to inform future studies in larger cohorts. All statistical tests were 2-sided, and analyses were performed using R (RStudio; version 2025.09.2 Build 418).

## Results

### Participation and Demographics

We enrolled 4 patients (3 men, 1 woman); the mean age was 54.3 (SD 8) years (range 48-66 years). Three participants were from the Hospital Provincial de Castellón, and 1 was from Hospital Dr Peset in Valencia.

Of the 4 participants, 3 (75%; patients 2-4) completed the 6-week reporting period, while 1 (25%; patient 1) did not, having discontinued participation after approximately 2 weeks due to unexpected premature death.

### Engagement With PROMs

Table 2 summarizes patient engagement with Lalaby-Glio across the 6-week study period. In total, 168 QoL questionnaires were completed: 22 QLQ-C30, 19 QLQ-BN20, and 127 daily status entries. The cumulative app use time was 240.21 minutes (approximately 4 hours), corresponding to a mean daily use of 1.8 (SD 2.7) minutes across all patients. The mean time required to complete the 3 weekly questionnaires was 7.2 (SD 3.3) minutes, with notable variation between individuals (range 5.43-9.32 minutes). Daily app use time differed between patients. Patient 3 showed the highest engagement, spending 95.3 minutes in the app (2.3, SD 3.5 minutes per day), followed by patient 2 with 77.7 minutes (1.9, SD 2.3 minutes per day), and patient 4 with 55.8 minutes (1.4, SD 2.1 minutes per day). Patient 1, who participated only during the first 2 weeks, used the app for 11.41 minutes in total (1.7, SD 3.0 minutes per day).

**Table 2:** Lalaby-Glio app use time and questionnaire completion per patient.

Measure	Patient 1	Patient 2	Patient 3	Patient 4	Total
Completed weeks (n/6)	2/6	6/6	6/6	6/6	20/24
Total time in app (minutes)	11.41	77.70	95.30	55.80	240.21
Time per day in app (minutes), mean (SD)	1.67 (3.00)	1.92 (2.30)	2.25 (3.50)	1.36 (2.1)	—^a^
Time per day in app (minutes), range	8.45-0.1	12.07-0.18	13.91-0.41	10.40-0.30	—
Time to complete 3 questionnaires (minutes), mean (SD)	8.45 (0)	7.53 (3.48)	9.32 (3.13)	5.43 (3.70)	—
QLQ-C30^b^ completed (n/7)	1/7	7/7	7/7	7/7	22/28
QLQ-BN20^c^ completed (n/7)	1/7	4/7	7/7	7/7	19/28
Daily status completed (n/42)	5/42	39/42	42/42	40/42	127/168

^a^Not applicable.

^b^QLQ-C30: Quality of Life Questionnaire-Core 30.

^c^QLQ-BN20: Quality of Life Questionnaire-Brain Neoplasm 20.

Figure 2 summarizes patient engagement with the Lalaby-Glio app, depicting individual patterns of daily and weekly questionnaire completion and the corresponding time spent in the app over the 6-week study period.

**Figure 2:**
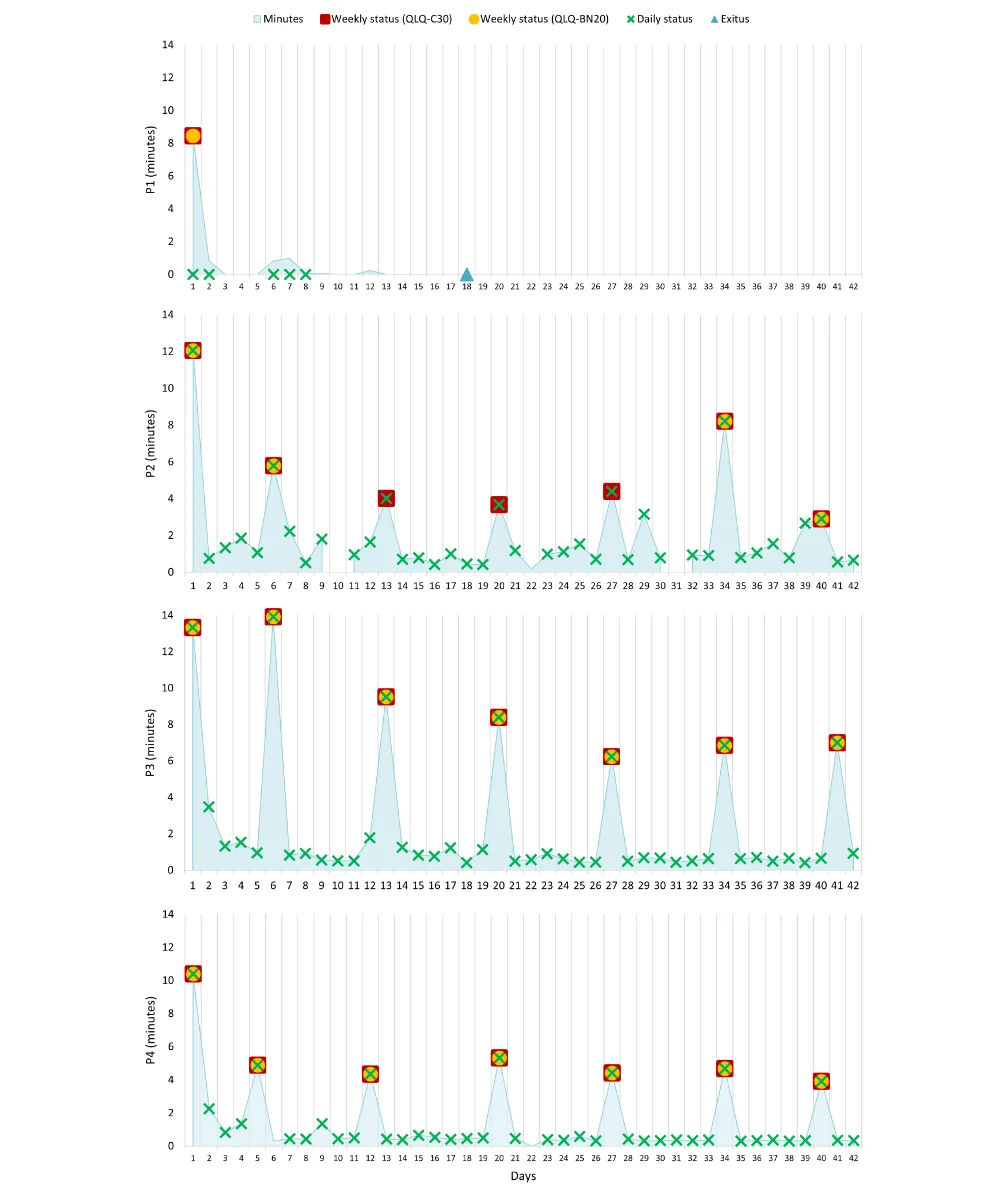
Patient reporting patterns for daily and weekly status and time spent in the Lalaby-Glio app. QLQ-BN20: Quality of Life Questionnaire-Brain Neoplasm 20; QLQ-C30: Quality of Life Questionnaire-Core 30.

### Patients’ Daily and Weekly Status

Daily status responses showed marked interpatient variability in symptoms and mobility during the monitoring period. Irritability and headache were mainly reported by patients 1 and 4, whereas the remaining patients reported few or no daily symptoms. Mobility limitations were also heterogeneous: patient 1 required a wheelchair during participation, patient 3 reported walking difficulties during weeks 3 and 4, and patients 2 and 4 did not report mobility problems. The mean reported sleep duration was 7.28 (SD 0.75) hours per night. Patient-level variability details are provided in Figures S1 and S2 in Multimedia Appendix 1.

Weekly EORTC QLQ-C30 (Figure 3) and QLQ-BN20 responses also revealed substantial heterogeneity across patients. Overall, patients 3 and 4 showed the most favorable QoL profiles, with high functional scores and low symptom burden, whereas patient 1 showed the poorest profile, characterized by severe functional impairment, high symptom burden, and very low global QoL. Patient 2 presented an intermediate and fluctuating profile over the 6-week period. Longitudinal trajectories further showed that patient 2 experienced early deterioration followed by partial stabilization, patient 3 maintained consistently high functioning and global QoL, and patient 4 generally showed high functional and global QoL, with a marked decrease in global QoL in week 2. Detailed patient-level functional, symptom, and brain tumor–specific questionnaire responses are provided in Figures S3-S9 in Multimedia Appendix 1.

**Figure 3:**
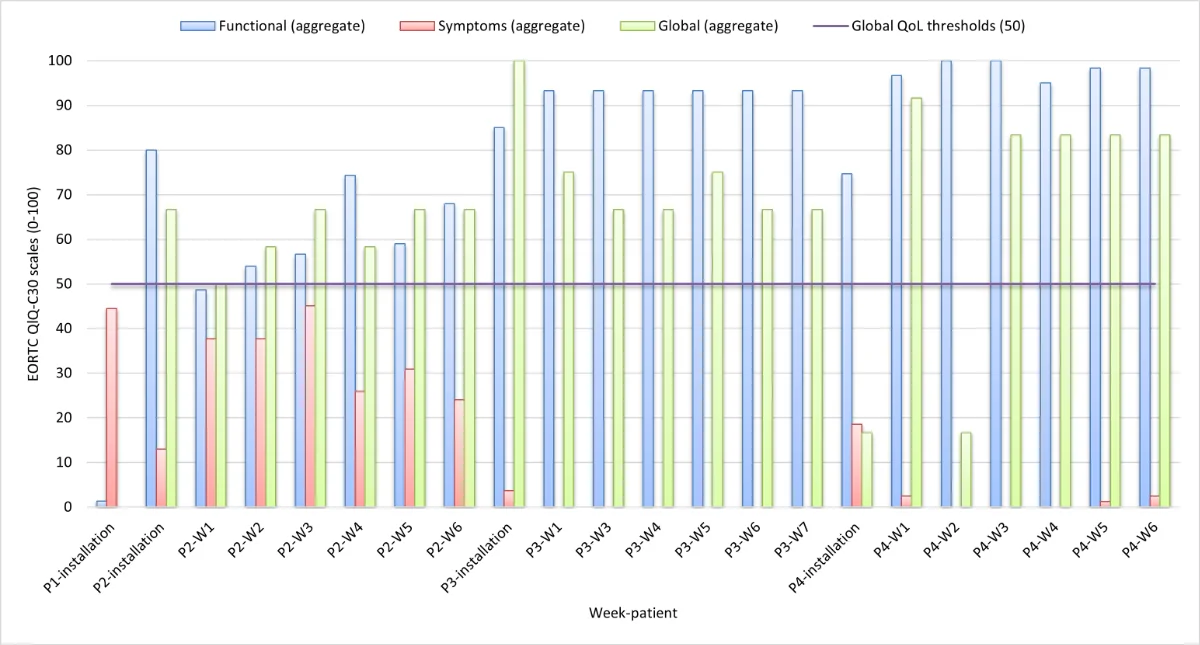
Longitudinal functional, symptom, and global quality of life (QoL) scores per patient over 6 weeks (European Organization for Research and Treatment of Cancer [EORTC] Quality of Life Questionnaire-Core 30 [QLQ-C30]). W: week.

### Automatic Data Collection via Smartphone Sensors

A total of 91.7 MB of passive sensor data were collected. Table 3 reports, for each patient, the weekly means (SD) and the number of recorded weeks (n) for all measures (steps, calls, data usage [download + upload], movement, sound intensity and frequency, light, distance, and screen time).

**Table 3:** Summary of mean values obtained from patients’ sensor data.

Measurement (weekly mean)	Patient 1, mean (SD); n	Patient 2, mean (SD); n	Patient 3, mean (SD); n	Patient 4, mean (SD); n
Steps	509.97 (493.35); 3	2937.08 (919.71); 6	9954.71 (1140.20); 6	6776.62 (2451.82); 6
Call (incoming and outgoing)	2.14 (2.35); 3	2.05 (1.30); 6	0.67 (0.52); 6	1.07 (0.85); 6
Data usage (MB)	976.46 (1015.94); 3	845.46 (204.45); 6	424.78 (63.77); 6	1159.37 (272.44); 6
Quantity of movement (m/s^2^)	0.99 (0.58); 3	9.81 (0.00); 6	6.32 (1.49); 6	0.54 (0.06); 6
Sound intensity (dB)	–99.93 (0.11); 3	–99.90 (0.05); 6	–99.82 (0.12); 6	–99.95 (0.04); 6
Sound frequency (Hz)	0.36 (0.59); 3	0.91 (0.25); 6	1.26 (0.92); 6	0.41 (0.35); 6
Light (lux)	88.69 (115.19); 3	222.50 (243.72); 6	142.02 (54.45); 6	No light sensor
Distance traveled (km)	0.27 (N/A^a^); 1	0.15 (0.33); 6	0.03 (0.07); 6	0.01 (0.00); 6
Lalaby-Glio app screen time (minutes)	2.38 (3.36); 2	1.45 (0.83); 6	1.81 (1.99); 6	0.91 (0.87); 6

^a^N/A: not available.

Figure 4 presents the sensors view of the Lalaby-Glio dashboard for patient 3, illustrating daily passive sensor data across the 6-week period. Corresponding dashboard visualizations for patients 1, 2, and 4 are provided in Figures S10-S12 in Multimedia Appendix 1.

**Figure 4:**
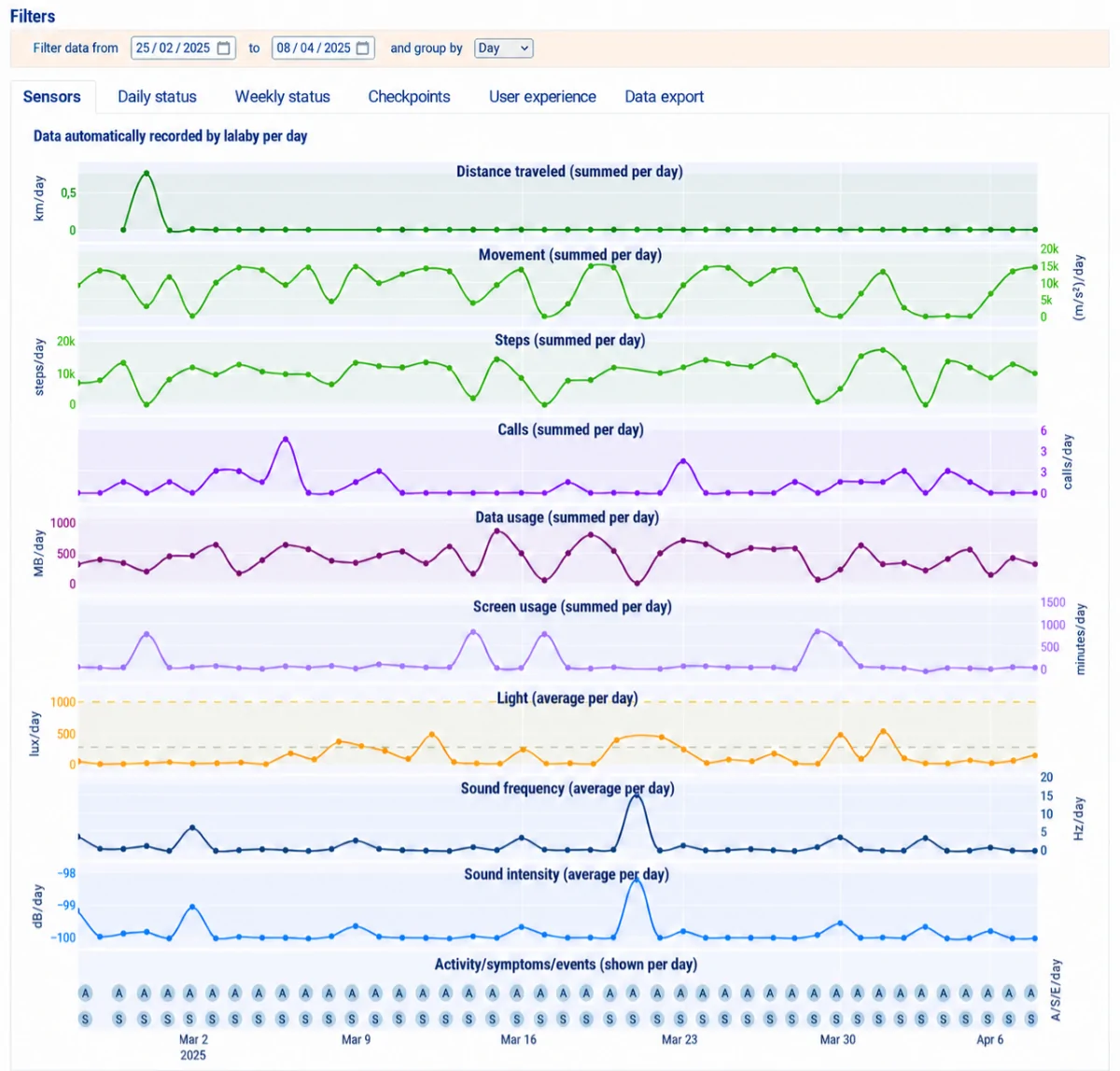
Sensors view of the Lalaby-Glio dashboard for patient 3 (daily, 6 weeks): the bottom panel displays daily status (walking ability and symptoms).

### Associations Between Global QoL and Passive Sensor Data

For ambulatory metrics (steps and movement), we excluded the wheelchair user (patient 1) to ensure biomechanical comparability. Under this specification, calls remained negatively and significantly associated with global QoL (ρ=−0.598; 95% CI −0.817 to −0.032; *P*=.01). Movement showed a negative, statistically significant association (ρ=−0.604; 95% CI −0.604 to −0.171; *P*<.001), whereas steps showed a positive but not significant association (ρ=0.392; 95% CI −0.428 to 0.671; *P*=.63). All other sensors exhibited weak or imprecise associations with CIs spanning 0.

When patient 1 was included, the calls finding was unchanged (negative and significant). Apparent associations for movement (ρ=−0.501; 95% CI −0.640 to 0.642; *P*=.29) and steps (ρ=0.486; 95% CI −0.352 to 0.791; *P*=.38) were not significant once clustering by patient was accounted for. Full comparison (with vs without patient 1) is provided in Table 4.

**Table 4:** Spearman rank correlations between sensor variables and global quality of life (patient-level bootstrap; 2-sided tests).

Variable	Spearman ρ (95% CI)^a^	*P* value (bootstrap)	Statistically significant
Calls (incoming and outgoing)	−0.598 (−0.817 to −0.032)	.01	Yes
Movement (m/s^2^)	−0.501 (−0.640 to 0.642)	.29	No
Movement (m/s^2^; patient 1 excluded)^b^	−0.604 (−0.604 to −0.171)	<.001	Yes
Steps	0.486 (−0.352 to 0.791)	.38	No
Steps (patient 1 excluded)^b^	0.392 (−0.428 to 0.671)	.63	No
Sound frequency (Hz)	−0.403 (−0.692 to 0.564)	.41	No
Sound intensity (dB)	−0.250 (−0.688 to 0.702)	.55	No
Light (lux)	−0.219 (−0.621 to 0.093)	.30	No
Lalaby-Glio app screen time (minutes)	−0.205 (−0.580 to 0.256)	.36	No
Distance traveled (km)	−0.184 (−0.687 to 0.400)	.56	No
Data usage (MB)	−0.014 (−0.785 to 0.541)	.65	No

^a^Bootstrap 95% CIs were estimated using the percentile method (2.5th and 97.5th percentiles).

^b^Patient 1 was excluded only from the analyses of steps and movement because wheelchair use makes these ambulatory metrics biomechanically noncomparable with those of ambulatory participants.

### Usability and UX

#### Overview

This section presents the experience outcomes of patients with GBM, followed by the usability evaluation with nonpatient users, to complement the study findings.

#### Patients With GBM

Regarding in-app UX ratings (scale 1-5), 1 rating per weekly submission was expected (24 in total); however, 32 ratings were recorded (133% of expected), with a mean rating of 3.7/5 (SD 0.97). Across the 6-week period, patient 2 consistently reported high UX (4/5), patient 3 remained stable at 3/5 after an initially higher score, and patient 4 showed a decline from 3/5 to 2/5 from week 4 onward; patient 1 provided a single rating (5/5) before discontinuation due to unexpected death. The evolution of patients’ UX ratings over time is shown in Figure S13 in Multimedia Appendix 1.

As part of the protocol, we contacted patient 4 after an alert was triggered in the dashboard due to an app rating below 3. This patient explained that they felt mobile phone use itself could negatively affect their condition and preferred direct contact with their oncologist rather than reporting via the app, although they did complete the 6 weeks of reporting.

During the installation interviews, we registered 4 mobile brands, all running Android versions 13 or higher, one of which lacked a light sensor. All patients rated the app’s simplicity and aesthetics as 5/5. Regarding the nature-based images, 2 patients felt these had no impact on their experience, while 2 reported a positive effect.

Using the “think aloud” technique, 6 improvements were identified, grouped into 2 categories:

- Modifications in the app: (1) adjusting font size for better readability, particularly with large text settings; (2) ensuring language consistency by using the first person throughout the questionnaires; (3) updating the QoL questionnaires by replacing the 1-4 labels with corresponding adjectives as per the standard EORTC QLQ-C30 and BN20 versions; and (4) replacing “@” and “a/o” with the patient’s gender as indicated in the registration form.
- Modifications in the dashboard: (1) adapting sensor visualization for phones lacking a light sensor or other hardware limitations and (2) configuring the dashboard for wheelchair users, introducing a new indicator such as “distance traveled” or “active minutes” to replace step count for patients using a wheelchair.

Detailed feedback from the interview data is provided in Tables S1 and S2 in Multimedia Appendix 1.

#### Nonpatient Users

In total, 103 healthy adults completed the survey (mean age 20.9, SD 1.4 years; range 20-30 years; 72/103, 69.9% women; 30/103, 29.1% men). Table 5 summarizes the results. Of these, 46 (44.7%) voluntarily installed and interacted with the app on their own phones; the remaining 57 (55.3%) evaluated the app using screenshot mock-ups of its sections. Only the installer group completed the SUS and reported usability problems. This group achieved a mean SUS score of 72.9 (SD 10.2; range 50-95), which is above the standard 68-point benchmark and indicates good/acceptable usability overall [35]. Subscale scores showed very strong learnability (items 4 and 10: 89.9/100, SD 13.6), indicating the app is easy to learn, while the broader usability dimension (items 1-3 and 5-9) was moderate to good (68.7/100, SD 11.5). Free-text responses on perceived errors in the app revealed several recurring issues. The most frequently mentioned problems concerned navigation and orientation between screens (n=20 comments), followed by visual design and information layout (n=11), lack of clear instructions or feedback (n=9), questionnaire length and configuration (n=7), and limited accessibility due to small buttons or icons (n=6). Less frequent but relevant issues included technical performance and passive data capture (n=2), insufficient oncology-specific or age-tailored content (n=2), limited visibility of patients’ own results (n=1), and the presence of unnecessary features (n=1). Detailed descriptions of each identified issue and the corresponding proposed solutions are provided in Table S3 in Multimedia Appendix 1.

**Table 5:** Nonpatient survey results: usability and aesthetic perceptions of the Lalaby-Glio app.

Measure	Result
Sample size, N	103
Age (years), mean (SD); range	20.9 (1.4); 20-30
Gender, n (%)	Women: 72 (69.9); men: 30 (29.1); prefer not to say: 1 (1)
App installed and used (own phone), n (%)	46 (44.7)
App assessed from screenshots, n (%)	57 (55.3)
Perceived simplicity (score 1-5), mean (SD)	3.93 (0.76)
Perceived aesthetics (score 1-5), mean (SD)	2.76 (0.88)
Perceived influence of nature on one’s own experience, n (%)	Positive: 83 (80.6); no influence: 20 (19.4); negative: 0 (0)
Perceived influence of nature on oncology patients’ experience, n (%)	Positive: 95 (92.2); no influence: 7 (6.8); negative: 1 (1)
Nature-based theme appropriate for oncology, n (%)	Yes: 94 (91.3); no: 9 (8.7)
SUS^a^ score (n=46), mean (SD)	72.9 (10.2)

^a^SUS: System Usability Score.

Across the full sample, perceived simplicity (Q1) was high (mean 3.9/5), while perceived visual aesthetics (Q2) was moderate (2.8/5). Most respondents considered nature-inspired imagery beneficial: in response to Q3, 80.6% (83/103) reported a positive influence on their own experience (20/103, 19.4% reported no influence; 0/103, 0% reported negative influence), and in Q4, 92.2% (95/103) judged that it would benefit oncology patients (7/103, 6.8% reported no influence; only 1/103, 1% reported negative influence). For Q5, most respondents (94/103, 91.3%) considered a nature-inspired design appropriate for oncology patients. Open-ended justifications for this judgment (Q6) were examined using quantitative content and bigram frequency analyses of the Spanish responses; the most frequent words and expressions (eg, tranquility, nature, calm, peace, conveys tranquility, conveys calm, and conveys peace) showed that participants consistently linked the design with serenity and emotional relief, suggesting that it helps convey calmness, reduce stress, and support a pleasant therapeutic environment. Detailed word frequencies and bigram frequencies (eg, the frequency with which pairs of consecutive words co-occur in the text) are presented in Tables S4 and S5 in Multimedia Appendix 1. Because only 9 participants indicated in Q5 that a nature-inspired design was not appropriate, their open-ended explanations in Q6 were reviewed manually: these respondents mainly questioned the relevance of nature to cancer, felt that digital nature cannot replace real nature, or saw the theme as too generic, and suggested alternative motifs (eg, cartoons, sports, travel, happiness, pets, and butterflies) or a more neutral, data-centered interface. Some were unsure, stating that suitability depends on implementation or that they did not understand the link between nature and a cancer app. Table S6 in Multimedia Appendix 1 provides the complete negative responses.

With regard to Q7 on appropriate themes, the top 20 words highlighted a clear preference for calm, nature-based imagery (Table S7 in Multimedia Appendix 1). Consolidating closely related terms, sea/ocean (sea + marine + oceanic; n≈29) emerged as the leading concept, followed by nature/natural (n≈15), animals (n=13), colors (n=12), and landscapes (n=9). Secondary cues included calm, sky, water, and art.

## Discussion

### Overview

The present study demonstrates the feasibility of remotely collecting both subjective and objective HRQoL data in adults with GBM using digital phenotyping by means of the Lalaby-Glio app. Over a period of 6 weeks, 3 patients scheduled to initiate standard Stupp treatment contributed continuous data, while 1 patient provided 2 weeks of data before death. Across participants, passive smartphone sensor data were captured without technical failures, and a total of 168 QoL questionnaires were completed (22 QLQ-C30, 19 QLQ-BN20, and 127 daily status entries). These findings demonstrate that Lalaby-Glio may facilitate sustained, unsupervised monitoring and generate a comprehensive dataset integrating daily and weekly PROMs and passive sensing, suitable for patient-level and cross-patient analyses.

The study confirmed a high level of adherence among the 3 patients who completed the study (Figure 2). Patient 3 submitted all expected questionnaires, patient 4 missed only 2 daily status reports, and patient 2 missed 2 QLQ-BN20 questionnaires and 3 daily status entries. Patient 1, who died during the follow-up period, had completed only the installation questionnaires and 5 daily reports. Overall, patients demonstrated a satisfactory comprehension of the mobile app and exhibited consistent engagement with it. This finding indicates that the reporting obligation is commensurate with the clinical and functional constraints frequently encountered in patients with GBM.

The mean duration of app tasks completed per day by patients was 1.8 (2.7) minutes. The weekly reporting process, which entailed completion of the QLQ-C30, QLQ-BN20, and daily status on the same day, required a mean of 7.2 (3.3) minutes, indicating a low and acceptable burden for this population. This result is slightly lower than the combined times previously reported in English-speaking populations with brain tumors, in which the QLQ-C30 and QLQ-BN20 required 5.2 and 3.1 minutes, respectively [36]. Notably, this was observed despite the inclusion of the daily status module in the same weekly reporting session. To the best of our knowledge, app-based completion times for these instruments have not been previously documented in Spanish-speaking patients with GBM, highlighting the novelty and relevance of these timing data in this feasibility study. Time is a critical resource in the care of patients with GBM [37]. During concomitant chemoradiotherapy and adjuvant temozolomide, patients already attend frequent in-person visits and undergo regular magnetic resonance imaging–based follow-up to monitor disease progression and treatment toxicity [38]. Adding additional face-to-face consultations to collect QoL information would further increase the burden on patients, caregivers, and clinical services. This may be difficult to implement in settings with limited resources. Lalaby-Glio enables remote, low-burden reporting, allowing this information to be captured without extending visit length or scheduling extra appointments. This represents a pragmatic advantage for integrating HRQoL monitoring into the care of patients with GBM.

From a clinical perspective, Lalaby-Glio successfully implemented the robust, widely validated EORTC QLQ-C30 and QLQ-BN20 measures of HRQoL in populations with brain tumors alongside a clinician-designed daily symptom questionnaire. This multimodal dataset enabled the longitudinal monitoring of HRQoL during standard Stupp treatment and revealed clear differences between patients. Daily status reports showed significant differences: patient 1 experienced high levels of irritability, headaches, and mobility impairment, whereas patient 3 reported no daily symptoms and maintained full mobility. Sleep monitoring revealed a mean sleep duration of 7.28 (SD 0.75) hours per night. There was a 1.5-hour difference between the 2 patients who consistently reported sleep; patient 3’s single entry likely reflects usability issues rather than insomnia (Figure S2 in Multimedia Appendix 1).

The weekly QLQ-C30 outcomes also revealed significant heterogeneity. Patient 1 exhibited the poorest functional and global QoL; patient 2 experienced persistent symptoms but remained above the clinical threshold for functioning. Patients 3 and 4 maintained a high QoL with only transient early symptoms. Brain tumor–specific symptoms on the QLQ-BN20 followed a similar pattern: patients 1 and 2 reported the greatest burden, patient 3 presented mainly with baseline symptoms, and patient 4 reported only mild, intermittent drowsiness.

In summary, these findings provide preliminary evidence that Lalaby-Glio is feasible and clinically informative for integrating a tailored daily status measure with validated PROMs to characterize QoL trajectories in GBM (Figures S3-S9 in Multimedia Appendix 1). To date, few mobile apps have implemented or evaluated the QLQ-C30 [39,40] or the QLQ-BN20 [41,42], positioning Lalaby-Glio as a novel and valuable tool for remote, structured HRQoL monitoring in this population.

From the perspective of passive data collection, our findings demonstrate that Lalaby-Glio can reliably capture smartphone sensor information in a fully automated manner. Across a total of 3 participants who were monitored over a period of 6 weeks and 1 participant who was monitored over a period of 3 weeks, the system collected a variety of data, including step counts, call activity (incoming and outgoing), data usage (received and sent), movement intensity (m/s^2^), sound intensity (dB), sound frequency (Hz), ambient light (lux), distance traveled (km), and in-app screen time (Table 3). The total amount of recorded sensor data was 91.7 MB, with no occurrence of technical failures. The dataset also exhibited substantial interpatient variability, as evidenced by patient 3 registering the highest step counts, patient 4 registering the greatest data use, and patient 1 contributing fewer observations. Moreover, patient 4 had no valid light sensor readings. This finding highlights the importance of accounting for hardware-related limitations in passive sensing studies. Future versions of Lalaby-Glio should include an initial hardware compatibility check during installation to identify unavailable or unsupported sensors, such as the light sensor. This would help inform patients and clinicians about which passive sensing features can be reliably collected for each device, manage expectations regarding the resulting digital phenotype, and improve the interpretation of missing or incomplete sensor-derived data.

We also examined the associations between these objective measures and the global QoL scale from the QLQ-C30, identifying significant negative correlations with call activity and movement intensity. These findings suggest that changes in digital and physical behavior may be associated with poorer perceived QoL. Increased call activity could reflect a greater need for social support, care coordination, or information seeking during periods of clinical deterioration, emotional distress, or treatment-related uncertainty. Alternatively, higher call activity may also represent an additional communication-related digital burden for patients reporting lower QoL. Similarly, higher movement intensity may not necessarily indicate better functioning but could reflect irregular, effortful, or distress-related activity patterns. Given the very small sample size, these associations should be interpreted cautiously as preliminary and hypothesis-generating, rather than as robust evidence of predictive modeling capacity or validated digital biomarkers. Overall, these preliminary findings suggest that validated subjective HRQoL indicators may be meaningfully linked to passive behavioral metrics. They also support further investigation into which sensor-derived features are most clinically relevant, how their correlations with QoL should be interpreted, and which features could be safely omitted to optimize data capture, storage, and processing.

From the perspective of usability, patients with GBM exhibited a commendable comprehension of the questionnaires and consistently perceived the app as straightforward to use. The nature-themed visual design was predominantly received positively or neutrally, with no instances of negative feedback. The mean overall app ratings were ≥3 throughout the study period, except for patient 4, whose rating decreased to 2 in the final 3 weeks. This observation may be explained by the patient’s preference for direct contact with their oncologist rather than reporting via the app, and it underscores the variability in how patients adopt and engage with digital health solutions. The case of patient 4 further illustrates that digital monitoring should not be understood as a substitute for the oncological relationship but as a complementary tool to support clinical awareness and communication. This finding also highlights digital burden as a clinically relevant factor, particularly in patients with advanced disease or fluctuating well-being. Accordingly, detecting early signs of patient burden is critical to enable timely de-escalation or pausing of app use, prioritize patient well-being, and maintain flexibility in clinical monitoring over continuous data capture. Future implementations could evaluate personalized PROM reporting schedules and patient-led “rest periods,” in which active PROM collection is temporarily paused while passive sensor data collection is maintained, with the option to resume app-based reporting when appropriate. These schedules could allow patients to temporarily activate or deactivate PROM reporting for predefined intervals, such as several weeks or 1 month, with an automatic alert sent to the clinical team. In addition, the app could automatically suggest a temporary rest period when negative UX ratings or other burden-related indicators are detected. Together, these adaptive strategies may help reduce patient burden while preserving longitudinal follow-up, continuity of objective monitoring, and patient engagement. In addition, usability testing identified 4 concrete areas for refinement, including better adaptation to larger font sizes and wording adjustments to improve comprehension and gender personalization. Although these refinements may appear minor, language consistency and gender-specific personalization are particularly important in this clinical context, where cognitive deficits, fatigue, or irritability may affect comprehension and tolerance of digital tools. Future versions should therefore ensure consistent first-person wording and replace neutral placeholders such as “@” or “a/o” with gender-specific language adapted to each patient.

Finally, the usability study with 103 healthy adults (mean age 20.9, SD 1.4 years; 72/103, 69.9% women) suggests that Lalaby-Glio is generally usable, easy to learn, and appropriate for oncology contexts. Of these, 46 (44.7%) installed the app and obtained a mean SUS score of 72.9 (SD 10.2), with high perceived simplicity but more moderate perceived aesthetics, while also highlighting areas for improvement, such as navigation, layout, clarity of instructions, and accessibility. Across the full sample, most participants perceived the nature-inspired design as beneficial for oncology patients (95/103, 92.2%) and appropriate for this context (94/103, 91.3%), although a small minority questioned its relevance and proposed alternative or more neutral themes. The high approval rating for the “tree of life” theme supports the use of biophilic, nature-inspired design elements in oncology apps to convey tranquility and calm. To the best of our knowledge, no study has validated the appropriateness of using a nature-based theme for an app for patients with GBM, a finding that lends relevance to the results presented herein.

### Strengths of the Study

The study’s major strengths include the multimodal nature of the data collected (daily PROMs, weekly PROMs, and passive sensing), the high level of adherence achieved, the time required being compatible with patients with GBM, and the overall usability and simplicity of the app, including a nature-inspired graphical theme that was judged appropriate for oncology. Notably, the study was conducted in patients undergoing active treatment for GBM, a population for whom traditional follow-up is often challenging. Lalaby-Glio demonstrates that continuous monitoring is both technically feasible and clinically informative in this context, and its ability to capture short-term changes in symptoms and functioning underscores its potential value for patient monitoring, clinical decision support, and supportive care.

### Limitations

This app-based study is inherently limited in that it excludes patients who do not own an Android smartphone or who are unable to use such devices confidently, which may result in a sample that is biased toward younger or more digitally literate individuals. A notable constraint pertains to the limited sample size, which is consistent with the findings of other feasibility studies integrating mobile apps into the care of patients with brain tumors. These studies have also reported small cohorts, a consequence of the fragility of this population (eg, Vinehealth app: N=4, including 2 GBM [41]; Cognition app: N=24, including 1 GBM [43]; Beiwe app: N=15 GBM [25]). The limited sample size imposes constraints on the statistical interpretation and external validity of the findings. The observed patterns may also be influenced by clinical heterogeneity, including variations in treatment phase and symptom severity. Furthermore, the quality of passive data may be influenced by device differences and variable phone-carrying behavior. Consequently, the statistical results should be regarded as preliminary and require validation in larger samples. Although adherence levels were high, concerns regarding the daily reporting burden suggest potential challenges for some patients.

### Implications and Future Directions

Despite these limitations, the study provides encouraging evidence supporting the use of Lalaby-Glio for continuous, noninvasive monitoring of QoL in GBM. Future work should focus on larger, multicenter cohorts; refining the daily symptom module; enhancing personalization; and adapting passive sensing indicators to different functional profiles. This is particularly relevant for wheelchair users, for whom distance traveled or wheelchair-based activity metrics may be more meaningful than step-based measures. These adaptations would improve the clinical interpretability of the dashboard and facilitate its integration into routine clinical workflows. Once embedded in clinical practice, Lalaby-Glio could enable early identification of deterioration, more timely interventions, and improved communication between patients and care teams. In the longer term, the digital phenotypes derived from combined PROMs and passive sensing offer the potential to develop predictive models of QoL based on objective, automatically collected data, which may evolve into robust digital biomarkers of QoL and functioning in neuro-oncology and support more personalized, adaptive models of care.

### Conclusions

Lalaby-Glio demonstrates that multimodal smartphone-based monitoring is feasible, acceptable, and capable of capturing clinically relevant signals in people living with GBM. By integrating PROMs with passive sensor data, the system offers a promising pathway for advancing noninvasive, real-time QoL monitoring in this vulnerable population.

## Appendix

Multimedia Appendix 1 Supplementary figures.

## Abbreviations

EORTC: European Organization for Research and Treatment of Cancer
GBM: glioblastoma multiforme
HRQoL: health-related quality of life
PROM: patient-reported outcome measure
QLQ-BN20: Quality of Life Questionnaire-Brain Neoplasm 20
QLQ-C30: Quality of Life Questionnaire-Core 30
QoL: quality of life
SUS: System Usability Scale
UX: user experience
