# Supplementary material for: Digital Phenotyping and Noninvasive Quality-of-Life Monitoring in Glioblastoma: Feasibility and Usability Study of the Lalaby-Glio App
Source: JMIR Form Res. 2026 Oct 1;10:e89765. doi: 10.2196/89765 (PMC13673174; doi:10.2196/89765)
Supplement: Supplementary file 1 [file formative_v10i1e89765_app1.docx]

**Multimedia Appendix 1.** Lalaby-Glio supplementary material.

## Daily status

Patient 1 reported irritability at different intensities (moderate, intense and very intense) and “a lot” of headache in week 1, whereas Patient 4 reported irritability in weeks 1 and 4 and “quite a bit” of headache in week 4. The remaining patients did not report any symptoms. With regard to mobility, Patient 1 required a wheelchair during both weeks of participation, and Patient 3 reported being able to walk but with difficulty in weeks 3 and 4. Patients 2 and 4 did not report any mobility difficulties (see Figure S1 in Multimedia Appendix).

On mean, patients slept 7.28 hours per night (SD 0.75). Patient 1 reported a mean of 7.25 hours of sleep per night (SD 0.35), with values ranging between 7 and 7.5 hours. Patient 2 showed slightly shorter and more variable sleep, with a mean of 6.75 hours (SD 0.50; range 6–7.4 hours). Patient 3 reported 6 hours of sleep on the only week with available data. In contrast, Patient 4 reported consistently long sleep durations, with values around 8 hours every week (mean 8.03 hours, SD 0.08) (see Figure S2 in Multimedia Appendix).

Entries/week

| Week-Patient | Mobility with wheelchair | Mobility alone but with difficulty | Irritability — mild | Irritability — moderate | Irritability — intense | Irritability — very intense | Headache — “quite a bit” | Headache — “a lot” |
| --- | --- | --- | --- | --- | --- | --- | --- | --- |
| W1-P1 | 4 | 0 | 0 | 1 | 1 | 1 | 0 | 1 |
| W1-P2 | 0 | 0 | 0 | 0 | 0 | 0 | 0 | 0 |
| W1-P3 | 0 | 0 | 0 | 0 | 0 | 0 | 0 | 0 |
| W1-P4 | 0 | 0 | 1 | 0 | 0 | 0 | 0 | 0 |
| W2-P1 | 1 | 0 | 0 | 0 | 0 | 0 | 0 | 0 |
| W2-P2 | 0 | 0 | 0 | 0 | 0 | 0 | 0 | 0 |
| W2-P3 | 0 | 0 | 0 | 0 | 0 | 0 | 0 | 0 |
| W2-P4 | 0 | 0 | 0 | 0 | 0 | 0 | 0 | 0 |
| W3-P1 | 0 | 0 | 0 | 0 | 0 | 0 | 0 | 0 |
| W3-P2 | 0 | 1 | 0 | 0 | 0 | 0 | 0 | 0 |
| W3-P3 | 0 | 0 | 0 | 0 | 0 | 0 | 0 | 0 |
| W3-P4 | 0 | 0 | 0 | 0 | 0 | 0 | 0 | 0 |
| W4-P1 | 0 | 0 | 0 | 0 | 0 | 0 | 0 | 0 |
| W4-P2 | 0 | 1 | 0 | 0 | 0 | 0 | 0 | 0 |
| W4-P3 | 0 | 0 | 0 | 0 | 0 | 0 | 0 | 0 |
| W4-P4 | 0 | 0 | 1 | 0 | 0 | 0 | 1 | 0 |
| W5-P1 | 0 | 0 | 0 | 0 | 0 | 0 | 0 | 0 |
| W5-P2 | 0 | 0 | 0 | 0 | 0 | 0 | 0 | 0 |
| W5-P3 | 0 | 0 | 0 | 0 | 0 | 0 | 0 | 0 |
| W5-P4 | 0 | 0 | 0 | 0 | 0 | 0 | 0 | 0 |
| W6-P1 | 0 | 0 | 0 | 0 | 0 | 0 | 0 | 0 |
| W6-P2 | 0 | 0 | 0 | 0 | 0 | 0 | 0 | 0 |
| W6-P3 | 0 | 0 | 0 | 0 | 0 | 0 | 0 | 0 |
| W6-P4 | 0 | 0 | 0 | 0 | 0 | 0 | 0 | 0 |

Figure S1. Mobility and symptoms reported in the Daily status Questionnaire by patient over the six-week reporting period

Hours

| Weeks | Patient 1 | Patient 2 | Patient 3 | Patient 4 |
| --- | --- | --- | --- | --- |
| Week 1 | 7.5 | 7.2 | 6 | 8.2 |
| Week 2 | 7 | 6 |  | 8 |
| Week 3 |  | 6.7 |  | 8 |
| Week 4 |  | 6.6 |  | 8 |
| Week 5 |  | 6.6 |  | 8 |
| Week 6 |  | 7.4 |  | 8 |

Figure S2. Mean Night-time Sleep (hours) by Week — All Patients

# **Weekly status**

## EORTC QLQ-C30

From a clinical perspective, the data collected show that, on patient means, Patients 3 and 4 exhibit the most QoL favorable profiles, characterized by very high aggregate Functional scores (92.1 and 94.7) and minimal aggregate Symptom scores (0.53 and 3.53). These patterns yield Global aggregate scores of 73.8 and 65.5, respectively. Patient 2 displays intermediate values (Functional 63.0; Symptoms 30.6; Global 61.9). In contrast, Patient 1 has the poorest profile, with very low Functional (1.33), high Symptoms (44.44), and a Global score of 0 (see Figure S3).

|  | Patient 1 | Patient 2 | Patient 3 | Patient 4 |
| --- | --- | --- | --- | --- |
| Functional | 1.33 | 62.95 | 92.14 | 94.71 |
| Symptoms | 44.44 | 30.6 | 0.53 | 3.53 |
| Global | 0 | 61.9 | 73.81 | 65.48 |

Figure S3. Mean Functional, Symptom, and Global Scale Scores per Patient over Six Weeks according to the EORT QLQ -C30 questionnaire.

|  | Patient 1 | Patient 2 | Patient 3 | Patient 4 |
| --- | --- | --- | --- | --- |
| PF_Physical (Q1–Q5) | 6.66667 | 64.7619 | 100 | 96.1905 |
| RF_Role (Q6–Q7) | 0 | 47.619 | 100 | 95.2381 |
| EF_Emotional (Q21–Q24) | 0 | 66.6667 | 98.8095 | 86.9048 |
| CF_Cognitive (Q20,Q25) | 0 | 73.8095 | 78.5714 | 97.619 |
| SF_Social (Q26,Q27) | 0 | 61.9048 | 83.3333 | 97.619 |

Figure S4. Functional subscales (EORTC QLQ-C30) per Patient (Mean over 6 Weeks)

Weekly trajectories (figure S5) revealed clear heterogeneity across patient. Patient 1 contributed only one weekly data point, showing high symptoms and extremely low functionality and global QoL. Patient 2 showed an early decline in week 1, with reduced functioning and global QoL alongside an increase in symptoms, followed by fluctuations and partial stabilization from weeks 3 to 6. Patient 3 maintained consistently high functioning and global QoL throughout the six weeks. Patient 4 presented high functional and global QoL along the treatment, although in week 2 a significant descries in global QoL was registered.

Score (0-100)

|  | PF_Physical (Q1–Q5) | RF_Role (Q6–Q7) | EF_Emotional (Q21–Q24) | CF_Cognitive (Q20,Q25) | SF_Social (Q26,Q27) | SF_Social (Q26,Q27) |
| --- | --- | --- | --- | --- | --- | --- |
| P1-Installation | 6.66667 | 0 | 0 | 0 | 0 | 0 |
| P2-Installation | 66.6667 | 66.6667 | 83.3333 | 100 | 83.3333 | 83.3333 |
| P2-W1 | 60 | 33.3333 | 50 | 50 | 50 | 50 |
| P2-W2 | 53.3333 | 33.3333 | 66.6667 | 66.6667 | 50 | 50 |
| P2-W3 | 66.6667 | 50 | 66.6667 | 66.6667 | 33.3333 | 33.3333 |
| P2-W4 | 80 | 66.6667 | 75 | 83.3333 | 66.6667 | 66.6667 |
| P2-W5 | 53.3333 | 33.3333 | 75 | 66.6667 | 66.6667 | 66.6667 |
| P2-W6 | 73.3333 | 50 | 50 | 83.3333 | 83.3333 | 83.3333 |
| P3-Installation | 100 | 100 | 91.6667 | 50 | 83.3333 | 83.3333 |
| P3-W1 | 100 | 100 | 100 | 83.3333 | 83.3333 | 83.3333 |
| P3-W3 | 100 | 100 | 100 | 83.3333 | 83.3333 | 83.3333 |
| P3-W4 | 100 | 100 | 100 | 83.3333 | 83.3333 | 83.3333 |
| P3-W5 | 100 | 100 | 100 | 83.3333 | 83.3333 | 83.3333 |
| P3-W6 | 100 | 100 | 100 | 83.3333 | 83.3333 | 83.3333 |
| P3-W7 | 100 | 100 | 100 | 83.3333 | 83.3333 | 83.3333 |
| P4-Installation | 73.3333 | 66.6667 | 66.6667 | 83.3333 | 83.3333 | 83.3333 |
| P4-W1 | 100 | 100 | 83.3333 | 100 | 100 | 100 |
| P4-W2 | 100 | 100 | 100 | 100 | 100 | 100 |
| P4-W3 | 100 | 100 | 100 | 100 | 100 | 100 |
| P4-W4 | 100 | 100 | 75 | 100 | 100 | 100 |
| P4-W5 | 100 | 100 | 91.6667 | 100 | 100 | 100 |
| P4-W6 | 100 | 100 | 91.6667 | 100 | 100 | 100 |

Figure S5. Longitudinal Functional Subscales Scores per Patient Over Six Weeks (EORTC QLQ-C30)

|  | Patient 1 | Patient 2 | Patient 3 | Patient 4 |
| --- | --- | --- | --- | --- |
| FA_Fatigue (Q10,Q12,Q18) | 66.6667 | 49.2063 | 0 | 12.6984 |
| NV_Nausea/Vomiting (Q14,Q15) | 0 | 9.52381 | 0 | 0 |
| PA_Pain (Q9,Q19) | 33.3333 | 30.9524 | 0 | 0 |
| DY_Dyspnoea (Q8) | 100 | 9.52381 | 0 | 4.7619 |
| SL_Insomnia (Q11) | 100 | 66.6667 | 4.7619 | 9.52381 |
| AP_Appetite loss (Q13) | 0 | 33.3333 | 0 | 0 |
| CO_Constipation (Q16) | 100 | 0 | 0 | 4.7619 |
| DI_Diarrhoea (Q17) | 0 | 38.0952 | 0 | 0 |
| FI_Financial difficulties (Q28) | 0 | 38.0952 | 0 | 0 |

Regarding the mean scores of the symptom. Patient 1 exhibited high levels of fatigue (66.7), dyspnoea (100), insomnia (100), pain (33.3), and constipation (100), indicating substantial symptom burden. Patient 2 showed moderate symptoms across fatigue (49.2), insomnia (66.7), appetite loss (33.3), diarrhoea (38.1), pain (30.9), and financial difficulties (38.1), reflecting partial impairment. Patient 3 had minimal symptom scores, with only a small level of Insomnia (4.8), indicating overall low symptom burden. Patient 4 experienced mild symptoms, fatigue (12.7), dyspnoea (4.8), insomnia (9.5), and constipation (4.8). These results highlighted inter-patient variability, with patients 1 and 2 experiencing higher symptom burden compared to patients 3 and 4 (see Figure S6 and S7).

Figure S6. Symptoms subscales (EORTC QLQ-C30) per Patient (Mean over 6 Weeks)

score (0-100)

| Week-Patient | FA_Fatigue (Q10,Q12,Q18) | NV_Nausea/Vomiting (Q14,Q15) | PA_Pain (Q9,Q19) | DY_Dyspnoea (Q8) | SL_Insomnia (Q11) | AP_Appetite loss (Q13) | CO_Constipation (Q16) | DI_Diarrhoea (Q17) | FI_Financial difficulties (Q28) |
| --- | --- | --- | --- | --- | --- | --- | --- | --- | --- |
| P1-Installation | 66.6667 | 0 | 33.3333 | 100 | 100 | 0 | 100 | 0 | 0 |
| P2-Installation | 33.3333 | 0 | 16.6667 | 0 | 33.3333 | 0 | 0 | 0 | 33.3333 |
| P2-W1 | 55.5556 | 0 | 50 | 0 | 66.6667 | 33.3333 | 0 | 66.6667 | 66.6667 |
| P2-W2 | 55.5556 | 16.6667 | 33.3333 | 33.3333 | 100 | 33.3333 | 0 | 33.3333 | 33.3333 |
| P2-W3 | 55.5556 | 33.3333 | 50 | 33.3333 | 66.6667 | 66.6667 | 0 | 66.6667 | 33.3333 |
| P2-W4 | 33.3333 | 16.6667 | 16.6667 | 0 | 66.6667 | 33.3333 | 0 | 33.3333 | 33.3333 |
| P2-W5 | 77.7778 | 0 | 33.3333 | 0 | 66.6667 | 33.3333 | 0 | 33.3333 | 33.3333 |
| P2-W6 | 33.3333 | 0 | 16.6667 | 0 | 66.6667 | 33.3333 | 0 | 33.3333 | 33.3333 |
| P3-Installation | 0 | 0 | 0 | 0 | 33.3333 | 0 | 0 | 0 | 0 |
| P3-W1 | 0 | 0 | 0 | 0 | 0 | 0 | 0 | 0 | 0 |
| P3-W3 | 0 | 0 | 0 | 0 | 0 | 0 | 0 | 0 | 0 |
| P3-W4 | 0 | 0 | 0 | 0 | 0 | 0 | 0 | 0 | 0 |
| P3-W5 | 0 | 0 | 0 | 0 | 0 | 0 | 0 | 0 | 0 |
| P3-W6 | 0 | 0 | 0 | 0 | 0 | 0 | 0 | 0 | 0 |
| P3-W7 | 0 | 0 | 0 | 0 | 0 | 0 | 0 | 0 | 0 |
| P4-Installation | 33.3333 | 0 | 0 | 33.3333 | 66.6667 | 0 | 33.3333 | 0 | 0 |
| P4-W1 | 22.2222 | 0 | 0 | 0 | 0 | 0 | 0 | 0 | 0 |
| P4-W2 | 0 | 0 | 0 | 0 | 0 | 0 | 0 | 0 | 0 |
| P4-W3 | 0 | 0 | 0 | 0 | 0 | 0 | 0 | 0 | 0 |
| P4-W4 | 0 | 0 | 0 | 0 | 0 | 0 | 0 | 0 | 0 |
| P4-W5 | 11.1111 | 0 | 0 | 0 | 0 | 0 | 0 | 0 | 0 |
| P4-W6 | 22.2222 | 0 | 0 | 0 | 0 | 0 | 0 | 0 | 0 |

Figure S7. Longitudinal Symptoms Subscales Scores per Patient Over Six Weeks (EORTC QLQ-C30)

## EORTC QLQ-BN20

Across specific brain tumor symptom means (EORTC QLQ-BN20), Patient 1 showed the highest overall symptom burden. Future uncertainty, motor dysfunction and bladder control were all maximal (100), with additional moderate problems related to communication (55.6) and headaches and visual disturbances (both 33.3). Drowsiness was also prominent (66.7), whereas seizures, hair loss, itchy skin and weakness of the legs were not reported. Patient 2 presented a mixed but clinically relevant symptom profile. Future uncertainty was moderate (47.9), with mild-to-moderate headaches (25), motor dysfunction (25) and communication difficulties (33.3). Drowsiness (50) and weakness of the legs (66.7) were the most pronounced symptoms, accompanied by some itchy skin (25) and minor bladder control problems (8.3). Patient 3 reported a comparatively lower symptom burden. future uncertainty was present but modest (28.6), with mild visual disorders (23.8), minor motor dysfunction (4.8), slight weakness of the legs (4.8) and some bladder control difficulties (23.8). Headaches, seizures, communication problems, drowsiness, hair loss and itchy skin were not reported. Patient 4 showed the least symptomatology, the only symptom reported was mild drowsiness (14.3), (see Figure S8 and S9).

|  | Patient 1 | Patient 2 | Patient 3 | Patient 4 |
| --- | --- | --- | --- | --- |
| FU_Future uncertainty (Q31,Q32,Q33,Q35) | 100 | 47.9167 | 28.5714 | 5.95238 |
| H_Headaches (Q34) | 33.3333 | 25 | 0 | 0 |
| VD_Visual disorder (Q36,Q37,Q38) | 33.3333 | 0 | 23.8095 | 0 |
| S_Seizures (Q39) | 0 | 0 | 0 | 0 |
| MD_Motor dysfunction (Q40,Q45,Q49) | 100 | 25 | 4.7619 | 0 |
| CD_Communication deficit (Q41,Q42,Q43) | 55.5556 | 33.3333 | 0 | 0 |
| Dr_Drowsiness (Q44) | 66.6667 | 50 | 0 | 14.2857 |
| HL_Hair loss (Q46) | 0 | 0 | 0 | 0 |
| IS_Itchy skin (Q47) | 0 | 25 | 0 | 0 |
| WL_Weakness of legs (Q48) | 0 | 66.6667 | 4.7619 | 0 |
| BC_Bladder control (Q50) | 100 | 8.33333 | 23.8095 | 0 |

Figure S8. Symptom subscales (EORTC QLQ-BN20) per patient (mean over six weeks)

score (0-100)

Figure S9. Longitudinal Symptoms Subscales Scores per Patient Over Six Weeks (EORTC BN20)

## **Automatic Data Collection via Smartphone Sensors**

Figure S10. Sensors view of the Lalaby-Glio dashboard for Patient 1. The line indicated the Exitus date and hour.

Figure S11. Sensors view of the Lalaby-Glio dashboard for Patient 2. The line indicated the Exitus date and hour.

Figure S12. Sensors view of the Lalaby-Glio dashboard for Patient 4. The line indicated the Exitus date and hour.

## **Usability and user experience**

Mean score (1-5)

|  | Patient 1 | Patient 2 | Patient 3 | Patient 4 |
| --- | --- | --- | --- | --- |
| Week 1 | 5 | 4 | 5 | 3 |
| Week 2 |  | 4 | 3 | 3 |
| Week 3 |  | 4 | 3 | 3 |
| Week 4 |  | 4 | 3 | 2 |
| Week 5 |  | 4 | 3 | 2 |
| Week 6 |  | 4 | 3 | 2 |

Figure S13. Patient Experience mean values over six weeks: UX Ratings (1–5)

Table S1. In-person installation CBM patient’s interview feedback (Part I).

| Patients | Mobile Brand | Android Version | Simplicity Rating (1-5) | Aesthetic Rating (1-5) | Nature (Positive, Negative, No Influence) |
| --- | --- | --- | --- | --- | --- |
| Patient 1 | Galaxy A15 | 15 | 5 | 5 | Positive |
| Patient 2 | Samsung A22 | 13 | 5 | 5 | Positive |
| Patient 3 | Ulefone Note 16 Pro | 13 | 5 | 5 | No Influence |
| Patient 4 | Samsung Galaxy A13 5G | 14 | 5 | 5 | No Influence |

Table S2. In-person installation CBM patient’s Think-aloud and observation feedback (Part II)

| **Patients** | **Observations** | **Recommendations** |
| --- | --- | --- |
| Patient 1 | - The patient had large text, and some questions were cut off by the white area of the image.  - Font size was reduced, but it was still difficult to read the text that wasn’t cut off.  - In the 30-questionnaire, "you" was used, and in the 20-questionnaire, "*usted*" was used. It should be unified to "you".  - The @ and a/o endings should be changed to match the patient’s gender.  - In the QoL-C30 questionnaire, is used, and in the BN20, a/o is used.  - The patient uses a wheelchair; how is step count calculated in this case?  - The patient was confused by the 1 to 7 quality of life rating scale because, in the previous questions, 1 meant "good" and 4 "bad", but in the last two questions, 1 meant "bad" and 7 "good", which caused confusion.  - In the C30 and BN20 questionnaires, the response options show numbers instead of adjectives, which confused the patient (1 instead of "not at all" and 4 instead of "much"). | - Ensure font size is appropriate for all settings and test readability.  - Unify language ("you" instead of "*usted*") in all questionnaires.  - Standardize gendered language or use consistent forms (e.g., "a/o" or gender-neutral terms).  - Clarify how step count is handled for wheelchair users.  - Maintain consistency in the quality of life scale (1 should mean "bad" and 7 "good" throughout).  - Replace numeric response options with descriptive adjectives or provide clear instructions for numeric scales. |
| Patient 2 | "Error when entering height; it records in meters (1.65) instead of centimeters." | - Correct the height entry error to ensure it’s recorded in centimeters. |
| Patient 3 | The patient was unclear on the first general quality of life question and did not fully understand that they had to select from 1 to 4. | - Clarify the instructions on the 1 to 4 scale for quality of life questions. |
| Patient 4 | The phone lacks a light sensor, so ambient light is not recorded. | - Consider adding a feature to handle phones without light sensors or inform patients of this limitation. |

Table S3. Summary of reported usability issues and explicitly proposed solutions for the Lalaby-Glio app (non-patients users)

| Error theme | Frequency (no. of comments*) | Explicit solutions proposed (English, UK) |
| --- | --- | --- |
| Navigation and orientation (current screen, moving between screens, back/exit buttons) | 20 | Use swipe gestures instead of coarse buttons to change screens; unify the layout of different screens so navigation feels more coherent; review navigation so it flows more smoothly; include a clear back arrow/button on screens where it is missing; configure the weekly-status screen so it opens only once per week and is disabled or hidden on other days; remove redundant “exit app” buttons, including the down-arrow on the home screen; provide a brief introductory explanation or short video tutorial so users know what happens after pressing each button. |
| Clarity of instructions, labels and feedback | 9 | Modify the sleep-hours screen so the answer is entered directly in the circle and clarify how the segments relate to hours (including >8h); add a short explanation of what the filling orange means; include small texts or explanations indicating what to do and what each icon is for; present information in a very direct way, especially for older adults; add a visible symbol, tick or color change when the daily status has been completed; highlight important parts of the questionnaire text; offer a brief introductory explanation or video tutorial. |
| Visual design, layout and graphics | 11 | Make the main screen more minimalist; redo the drawings and overall visual style; adopt a more dynamic aesthetic (typography and graphic quality); make the interface more visually appealing and stimulating for oncology patients, with more engaging images to match words and drawings; unify screens so that the visual style is consistent; reduce the visual impact of decorative elements such as the growing tree if they are only decoration; make the questionnaire interface more lively and dynamic by changing illustrations as the user progresses; make graphs easier to read. |
| Accessibility of controls (button/icon size, contrast) | 6 | Use an alternative control for some questions, such as a slider scale to adjust pain more precisely; prioritize larger buttons and clearer indicators; improve accessibility with larger buttons, the option of voice control and compatibility with screen readers; increase the size of icons, particularly for older adults. |
| Questionnaire length, burden and configuration | 7 | Use a slider-type control to provide more flexible responses in tests (e.g., for pain level); configure the weekly-status questionnaire so it opens only once a week; provide a graphical completion indicator (tick/color change) for the daily status; if the decorative tree is not informative, reduce its size so it distracts less; arrange many questions together or by categories on a scrollable screen; make the questionnaire interface more dynamic with changing illustrations; synthesize or reduce the number of quality-of-life questions. |
| Technical performance and passive data capture | 2 | Optimize permission management and background operation so passive data (e.g., movement, voice) continue to be recorded even when the operating system restricts background activity; optimize overall app performance and navigation so sections load faster and respond better to taps. |
| Content coverage and tailoring to oncology/age groups | 2 | Include configurable age profiles (children, adolescents, adults) with adapted language, visual style and types of exercises; add new content focused on emotional management, preparation for treatments, sleep improvement and support for caregivers and children, tailored to the oncology context. |
| Visibility of patients’ own results | 1 | Add screens that allow patients to see at least part of their results in a simple way that is useful but not overwhelming. |

Table S4. Word-frequency analysis of open-ended responses on the appropriateness of a nature-inspired design for oncology patients

| Spanish term | English (UK) term | n |
| --- | --- | --- |
| *tranquilidad* | tranquility | 38 |
| *naturaleza* | nature | 35 |
| *transmite* | conveys | 34 |
| *calma* | calm | 27 |
| *paz* | peace | 21 |
| *pacientes* | patients | 17 |
| *puede* | may | 17 |
| *app* | app | 11 |
| *bienestar* | wellbeing | 9 |
| *temática* | theme | 9 |
| *además* | moreover | 8 |
| *paciente* | patient | 8 |
| *sensación* | feeling | 8 |
| *positivo* | positive | 7 |
| *transmitir* | to convey | 7 |
| *aporta* | contributes | 6 |
| *ayudar* | to help | 6 |
| *creo* | I think | 6 |
| *emocional* | emotional | 6 |
| *esperanza* | hope | 6 |
| *estrés* | stress | 6 |
| *oncológicos* | oncology | 5 |
| *positiva* | positive | 5 |
| *relajación* | relaxation | 5 |
| *salud* | health | 5 |
| *vida* | life | 5 |
| *ambiente* | environment | 4 |
| *ayuda* | help | 4 |
| *estética* | aesthetics | 4 |
| *experiencia* | experience | 4 |

Table S5. Most frequent bigrams in open-ended responses on the appropriateness of a nature-inspired design for oncology patients

| Spanish bigram | English (UK) bigram | n |
| --- | --- | --- |
| *transmite tranquilidad* | conveys tranquility | 11 |
| *naturaleza transmite* | nature conveys | 11 |
| *transmite calma* | conveys calm | 11 |
| *transmite paz* | conveys peace | 8 |
| *paz tranquilidad* | peace and tranquility | 7 |
| *calma tranquilidad* | calm and tranquility | 6 |
| *pacientes oncológicos* | oncology patients | 5 |
| *puede ayudar* | can help | 5 |
| *bienestar emocional* | emotional wellbeing | 4 |
| *sensación tranquilidad* | feeling of tranquility | 4 |
| *naturaleza puede* | nature can | 3 |
| *puede transmitir* | can convey | 3 |

Table S6. Original Spanish Q6 comments for “No” responses and their English translations

| ID | Q6 reason (Spanish) | Q6 reason (English) |
| --- | --- | --- |
| 1 | Porque la naturaleza digital no puede suplir la falta de contacto con la naturaleza real que tienen los pacientes de un hospital. Y puede resultar una fuente de añoranza y tristeza. | Because digital nature cannot make up for the lack of contact with real nature that hospital patients have, and it may become a source of longing and sadness. |
| 2 | Porque es un tema que sí que es verdad que abarca realmente todo, pero pienso que quizás otros temas más entretenidos o divertidos ayudarían a esas personas a conseguir huir de la monotonía y hacer uso de la aplicación de manera distinta. | Because it is a theme that does indeed cover almost everything, but I think that other, more entertaining or fun themes might help these people escape monotony and use the app in a different way. |
| 3 | No tiene nada que ver la naturaleza con la oncología y los diseños podrían mejorarse. | Nature has nothing to do with oncology and the designs could be improved. |
| 4 | No veo mucha relación entre la naturaleza y una enfermedad, las apps de hospitales por ejemplo no tienen mucha relación con la naturaleza por lo que tampoco lo veo muy necesario. | I do not see much connection between nature and an illness; hospital apps, for example, are not very related to nature, so I do not see it as very necessary either. |
| 5 | Creo que se podría representar con otro motivo natural que tuviera una connotación más relacionada con la enfermedad y que no fuera un naranjo. | I think it could be represented with another natural motif that has a connotation more closely related to the illness and that is not an orange tree. |
| 6 | No sé en qué puede ser mejor la naturaleza para un paciente oncológico, no le veo una relación directa. | I do not know in what way nature can be better for an oncology patient; I do not see a direct relationship. |
| 7 | Es un tema genérico sin más, no le veo especial relevancia para este uso. | It is just a generic theme; I do not see any particular relevance for this use. |
| 8 | Sin más, no aporta nada y no guarda relación. | Nothing more; it adds nothing and is not related. |
| 9 | No entiendo el vínculo de la naturaleza con una aplicación para el cáncer. | I do not understand the link between nature and an app for cancer. |

Table S7. Top-20 word-frequency analysis of open-ended responses on suggested alternative themes for the Lalaby app in oncology contexts

| Word | n |
| --- | --- |
| Sea | 18 |
| Animals | 13 |
| Colors | 12 |
| Nature | 11 |
| Landscapes | 9 |
| Marine | 7 |
| Calm | 6 |
| Sky | 5 |
| Water | 4 |
| Art | 4 |
| Background | 4 |
| Oceanic | 4 |
| Natural | 4 |
